# The Development of the Innovative Synthesis Methodology of Albumin Nanoparticles Supported by Their Physicochemical, Cytotoxic and Hemolytic Evaluation

**DOI:** 10.3390/ma14164386

**Published:** 2021-08-05

**Authors:** Sonia Kudłacik-Kramarczyk, Anna Drabczyk, Magdalena Głąb, Paweł Gajda, Anna Czopek, Agnieszka Zagórska, Anna Jaromin, Jerzy Gubernator, Agnieszka Makara, Bożena Tyliszczak

**Affiliations:** 1 Department of Materials Science, Faculty of Materials Engineering and Physics, Cracow University of Technology, 37 Jana Pawła II Av., 31-864 Krakow, Poland; 2 Department of Sustainable Energy Development, Faculty of Energy and Fuels, AGH University of Science and Technology, 30 Mickiewicza Av., 30-059 Krakow, Poland; pgajda@agh.edu.pl; 3 Department of Medicinal Chemistry, Faculty of Pharmacy, Jagiellonian University Medical College, 9 Medyczna St., 30-688 Krakow, Poland; anna.czopek@uj.edu.pl (A.C.); agnieszka.zagorska@uj.edu.pl (A.Z.); 4 Department of Lipids and Liposomes, Faculty of Biotechnology, University of Wroclaw, 14a Joliot-Curie St., 50-383 Wroclaw, Poland; anna.jaromin@uwr.edu.pl (A.J.); jerzy.gubernator@uwr.edu.pl (J.G.); 5 Faculty of Chemical Engineering and Technology, Cracow University of Technology, 24 Warszawska St., 31-155 Krakow, Poland; agnieszka.makara@pk.edu.pl

**Keywords:** albumin spheres, nanoparticles, salt-induced protein precipitation, salting-out agent, hemolytic activity, MTT reduction assay, TEM imaging, spectroscopic analysis

## Abstract

Many studies are being performed to develop effective carriers for controlled cytostatic delivery wherein albumin is a promising material due to its tendency to accumulate near cancer cells. The novelty of this work involves the development of the synthesis methodology of albumin nanoparticles and their biological and physicochemical evaluation. Albumin particles were obtained via the salt-induced precipitation and K_3_PO_4_ was used as a salting-out agent. Various concentrations of protein and salting-out agent solutions were mixed using a burette or a syringe system. It was proved that the size of the particles depended on the concentrations of the reagents and the methodology applied. As a result of a process performed using a burette and 2 M K_3_PO_4_, albumin spheres having a size 5–25 nm were obtained. The size of nanospheres and their spherical shape was confirmed via TEM analysis. The use of a syringe system led to preparation of particles of large polydispersity. The highest albumin concentration allowing for synthesis of homogeneous particles was 2 g/L. The presence of albumin in spheres was confirmed via the FT-IR technique and UV-Vis spectroscopy. All samples showed no cytotoxicity towards normal human dermal fibroblasts and no hemolytic properties against human erythrocytes (the hemolysis did not exceed 2.5%).

## 1. Introduction

The main purpose of both pharmacology and pharmacokinetics is to achieve the best therapeutic effect. Firstly, main attention was paid to the development of new active substances. Now, research is mainly focused on increasing the therapeutic effectiveness of drugs concerning the changes in the drug delivery methodology [1]. Innovative methods of drug delivery mainly include the application of the appropriate carriers [2]. Most often, such carriers are based on inorganic materials as well as on natural and synthetic polymers, wherein the natural ones are particularly useful due to their biodegradability and non-toxicity [3,4]. A particularly valuable group of natural polymers are proteins such as albumin, one of the main tasks of which is to transport substances such as free fatty acids, hormones, vitamins and drugs [5].

Albumin is the most abundant plasma protein. There are different types of this protein wherein the ones which are widely applicable for biomedical purposes are human serum albumin (HSA), bovine serum albumin (BSA) and the egg white one [6]. Albumin is highly available [7], biodegradable [8] and biocompatible [9]. Importantly, this protein has a tendency to accumulate in inflamed tissues and in cancer tissues [10]. The mentioned characteristics indicate that albumins, both human and animal, are promising materials for preparation of drug carriers.

Currently, albumin-based drug carriers are considered for application in the treatment of diabetes, infectious diseases and cancers [11]. Albumin nanoparticles incorporated with cytostatic were designed by Onafuye et al. In the research, they presented nanoparticles based on human serum albumin obtained via the desolvation process. They were stabilized by the crosslinking of free amino groups present in the structure of albumin. Such obtained nanoparticles were incorporated with doxorubicin. The anticancer activity of these nanomaterials was verified towards the neuroblastoma cell line UKF-NB-3. It was proved that albumin may be applied as a specific agent for the delivery of cytostatics [12]. Albumin nanoparticles containing doxorubicin were also a research subject of the investigations of Kayani et al. They showed an innovative synthesis methodology of preparation of spheres via the desolvation process performed using dimethyl sulfoxide (DMSO). As a result, doughnut-shaped BSA nanoparticles showing narrow size distribution and homogeneity were obtained [13]. In turn, Taneja et al. developed a methodology of combining HSA nanoparticles with irinotecan. For this purpose, they used a hydrophilic non-ionic surfactant (polysorbat 80) thus forming a protein-polysorbat 80 complex. Such formed complex showed higher affinity to the drug applied. The drug entrapment inside the nanoparticles was confirmed via a differential scanning calorimeter, Fourier transform infrared spectroscopy and fluorescent spectroscopy. Based on in vitro drug release studies it was reported that the release of irinotecan was two-phase. Importantly, the results obtained indicated the rapid release of this active substance at the beginning of the study (20–25%) which was probably related to the weak bond between the drug and the carrier. Next, during the second phase of the study a long-term release (to 24 h) of the drug was observed. Finally, it was proved that the whole drug was released from the analyzed nanoparticles [14]. Albumin-based drug carriers were also investigated by Wang et al. [15], and Fattahian Kalhor et al. [16].

Albumin nanoparticles may be obtained via such techniques as desolvation [17], emolation [18], thermal gelation [19], nanospray drying [20] and nanoparticle albumin-bound (NAB) technology [21]. Due to the relatively mild conditions required as well as the simple methodology the desolvation process is one of the most frequently applied methods of protein nanoparticles preparation. It includes the addition of such desolving agents as ethanol or acetone to the protein solution [22]. The desolvation process aimed at preparation of albumin nanoparticles was used e.g., by Weber et al. Their synthesis methodology included the preparation of an aqueous protein solution followed by the dropwise addition of ethanol. After the precipitation, the nanoparticles obtained were stabilized via the crosslinking using 2.7% aldehyde solution [23]. The protein desolvation process was also presented by Das et al. [24] and Jahanban-Esfahlan et al. [25]. Nonetheless, in presented investigations the crosslinking of unstable particles showing a high tendency to agglomerate was necessary.

An interesting solution which allows to obtain stable particles with small sizes is the protein salting out (named also as salt induced protein precipitation or salt fractionation). This method is characterized by such advantages as low cost, high yield as well as the methodology simplicity. Furthermore, this method does not require the application of any toxic solvents and the selection of the adequate concentration of the salting out agent allows to control the size of the nanoparticles obtained [26]. Such a methodology was proposed via Lammel et al. who prepared silk fibroin particles as a result of the salting out process proceeding with the use of potassium phosphate as a salting out agent. The aqueous protein solution was mixed with the potassium phosphate in a volume ratio 1:5. Next, the particles obtained were stored for 2 h at 4 °C, centrifuged and washed with distilled water. Performed dynamic light scattering (DLS) confirmed the preparation of silk fibroin particles with controlled sizes within the range 500 nm–2 mm [27].

Due to the unique properties of albumin-based drug carriers, they constitute materials which constitute a main research subject of many investigations. Nevertheless, to our knowledge there are no reports concerning the preparation of albumin-based carriers in the protein salting out method. This process seems to be a promising method, therefore the main purpose of the presented research is to develop an innovative synthesis methodology of albumin nanoparticles. Here, main attention was paid to the selection of appropriate synthesis conditions. Various salting-out agents as well as various albumin solution concentrations and salting-out agent concentrations were investigated. In order to verify the effectiveness of the performed syntheses, such techniques as Fourier transform infrared (FT-IR) spectroscopy and UV-Vis spectrophotometry were applied. Next, the size and the dispersity of the albumin particles obtained were determined via the DLS method. Considering the application of the albumin particles for biomedical purposes, particularly as drug carriers, the essential part of the investigations was to evaluate their cytotoxicity towards normal human dermal fibroblasts (NHDF) and the hemolytic properties against human erythrocytes.

## 2. Materials and Methods

### 2.1. Materials

Albumin (albumin egg powder, max. 8% moisture), hydrochloric acid (35–38%, d = 1.19 g/mL), potassium dihydrogen phosphate and calcium chloride were purchased in Avantor Performance Materials Poland S.A. (Gliwice, Poland). Potassium phosphate, phosphate buffered saline (PBS, form: Tablet) and tris (hydroxymethyl) aminomethane (≥99.8%, ACS reagent) were bought in Merck (Darmstadt, Germany). Alpha-MEM and the normal human dermal fibroblast cell line (NHDF) were purchased in Lonza (Lonza, Warsaw, Poland). Fetal bovine serum (FBS) was from Biomedia (EuroClone by Biomedica, Piaseczno, Poland). MTT (3-(4,5-dimethylthiazol-2-yl)-2,5-diphenyltetrazolium bromide) was from Sigma-Aldrich (Poznan, Poland). All reagents were applied without further purification.

### 2.2. The Development of the Synthesis Methodology of Albumin Particles

The salting out process was selected for preparation of albumin-based carriers. Firstly, a number of syntheses were performed to identify the most favorable process conditions. Tris-HCl buffer with pH = 7 was selected as a solvent for albumin. This buffer is widely used e.g., in biochemistry or in molecular biology during the isolation and storage of RNA and DNA [28,29]. The preparation of the buffer included the preparation of 10 mM Tris solution and its subsequent acidification using hydrochloric acid to obtain the adequate pH of the final solution. According to the literature reports, the salting out process proceeds most effectively near the isoelectric point of the protein. Then, such a protein shows the lowest solubility and precipitates most easily from the solution [30]. The isoelectric point for ovalbumin occurs at pH approx. 4.8 [6], so Tris-HCl buffer solution with pH = 4.8 was also used in the particle synthesis. Next, various salting-out agents were applied. For example, Dumetz et al. during the investigations concerning the interactions of proteins in various salt solutions proved that the effective salting-out agent for ovalbumin is potassium phosphate monobasic [31]. Therefore, this salt was applied as a salting out agent in the research presented in this paper. Next, potassium phosphate was also used to determine the impact of potassium ions on the protein salting out process. The third compound which was tested was calcium chloride—an inorganic salt which was also determined to be an effective salting-out agent [32,33].

At the beginning, the syntheses were conducted at a constant protein solution concentration, i.e., 2 g/L, wherein the parameter which was changed was the pH of the Tris-HCl buffer solution in which albumin was dissolved. Moreover, various concentrations of the salting-out agents (i.e., 1 and 2 mol/L) mentioned previously were also checked.

The synthesis methodology of the albumin particles involved the preparation of the adequate solutions followed by dropping the albumin solution into the salting-out agent solution. The solutions were mixed in a 1:1 volume ratio using two methodologies, i.e., by means of a burette or via a syringe system.

In the first mentioned method, the solutions were mixed using a burette (SOLARIUS automatic burette, m-u-t GmbH, Wedel, Germany) wherein the speed of the dropwise addition was 1 drop/s. The protein solution was dropped into the salting-out agent solution and the solutions were mixed using a magnetic stirrer (IKA C-MAG HS 7 magnetic stirrer, IKA^®^ Poland Sp. z o.o., Warsaw, Poland, 80 rpm, room temperature). In order to remove the remaining amount of the salting-out agent, the resulting suspensions were subjected for 15 min to the centrifugation process (laboratory plasma centrifuge TD4C, 1500 rpm, room temperature) and the sediment obtained was next suspended in a PBS buffer. Selected compositions were analyzed using the DLS method. Such an investigation allowed to determine the type of the salting-out agent applied and the pH of the Tris-HCl buffer used on the size and dispersity of obtained albumin particles. Subsequently, two synthesis routes were conducted, i.e., in the first one the albumin particles were obtained using a constant concentration of albumin and various concentrations of potassium phosphate, wherein the second route included syntheses performed using a constant salting-out agent concentration and various albumin concentrations.

The second methodology applied to mix the albumin solution with the salting-out agent solution involved their dosing via a syringe system. The first syringe contained the albumin solution, wherein in the second one the salting-out agent solution was placed. Then, both solutions were pressed via the syringes into a mixing zone where the protein salting-out process proceeded. Next, as it was in the previous methodology, the albumin particle suspension obtained was subjected to the centrifugation process and suspended in the PBS buffer. All obtained compositions were analyzed via FT-IR spectroscopy and UV-Vis spectrophotometry to verify the preparation of albumin particles. Furthermore, the DLS technique allowed to determine their size and dispersity. Selected materials were also subjected to the investigations on their cytotoxicity and hemolytic properties.

**Figure 1:**
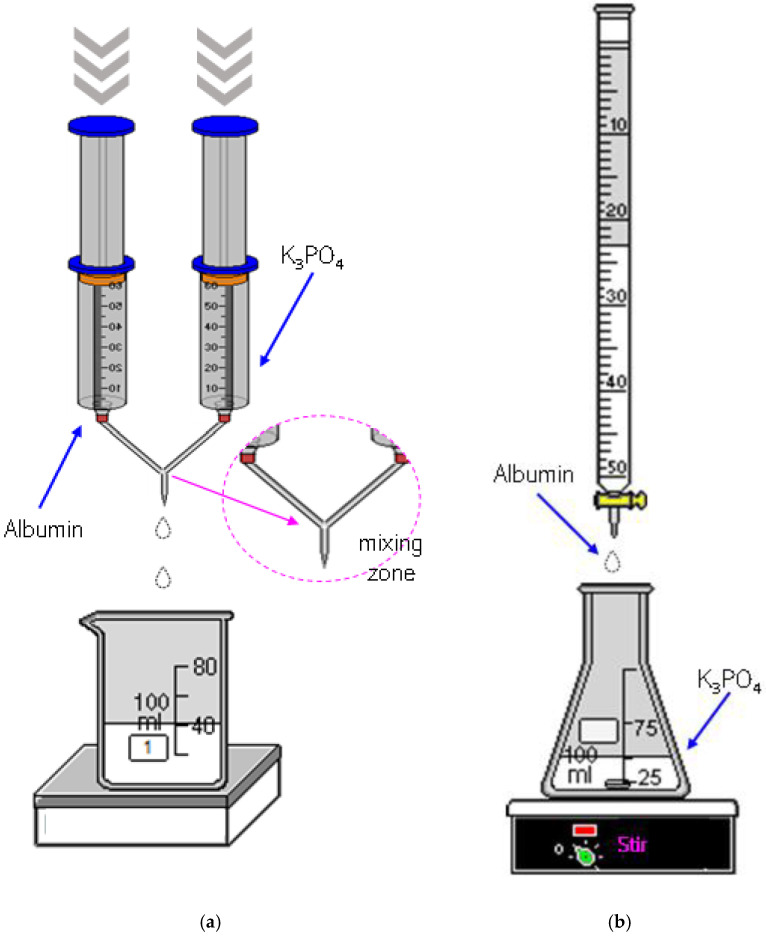
The scheme of the albumin-based carriers synthesis via a syringe system (**a**) and using a burette (**b**).

Below in Figure 1 the schemes of both methods used to prepare the albumin-based carriers are shown.

The particles obtained were washed with distilled water to neutral pH, suspended in the PBS buffer and subjected to numerous analyses; the methodologies applied are described below in Section 2.3.

### 2.3. Measurement Methodology of the Albumin-Based Particles

#### 2.3.1. Analysis of the Sizes of Albumin Particles via Dynamic Light Scattering (DLS) Technique

Study was performed to determine the size of the albumin particles, wherein main attention was paid to evaluate the impact of the methodology applied, the concentration of the reagent used during the synthesis as well as the pH of the reaction environment on this parameter. Measurement was conducted at room temperature using a Zetasizer Nano ZS Malvern (Malvern Panalytical Ltd., Malvern, UK). 

#### 2.3.2. Morphological Analysis Using Transmission Electron Microscopy (TEM)

The assessment of albumin nanoparticles was performed using a TESLA BS 540 (TESCAN, Brno, Czech Republic) transmission electron microscope. Before the measurement, the sample was diluted, introduced onto a copper grid and dried at room temperature. Finally, the nanoparticles were stained with 2% uranyl acetate and dried again.

#### 2.3.3. Analysis of the Chemical Structure of Particles Obtained Using Fourier Transform Infrared (FT-IR) Spectroscopy

Fourier transform infrared (FT-IR) spectroscopy allowed to identify the functional groups in the tested particles. The analysis was performed by means of the Nicolet iS5 Thermo Scientific (Thermo Fisher Scientific, Waltham, MA, USA) spectrometer. In order to perform a spectroscopic analysis, a drop of each prepared suspension of albumin particles (both the ones obtained using a syringe system and a burette) was placed on a diamond ATR crystal. The measurement range was 4000–550 cm^−1^ wherein the spectra were recorded at 4.0 cm^−1^ resolution.

#### 2.3.4. Studies on the Optical Properties of Albumin Particles via UV-Vis Spectrophotometry

Optical properties of albumin particles were verified via UV-Vis spectroscopy. The study made it possible to identify the chemical structures absorbing light from the UV-Vis range. The measurements were performed at room temperature and using the ThermoScientific Evolution 220 UV-Vis spectrometer (Thermo Fisher Scientific, Waltham, MA, USA). Results of the analysis are presented in the Supplementary File. Obtained UV-Vis spectra are shown in Figure S4.

#### 2.3.5. Assessment of Cytotoxicity

- MTT reduction assay

NHDF (Normal Human Dermal Fibroblasts) cells were cultured as previously described [34]. Different doses of albumin nanoparticles prepared in PBS (sample 1–6) were added to each well, then mixed and subsequently incubated for 48 h. Cell viability was determined using the MTT assay [35]. Results were expressed as the percentage of survival cells with respect to the control (not treated cells).

- Assessment of the hemolytic activity

The hemolytic properties were performed according to the previously published method [36]. The study protocol was approved by the Bioethics Commission at the Lower Silesian Medical Chamber (1/PNHAB/2018). Albumin nanoparticles (samples 1–6) prepared in PBS were added in a volume corresponding to the final concentration of 10 µL/mL in the test tube and incubated with human erythrocytes in PBS for 30 min at 37 °C. Subsequently, samples were centrifuged and the absorbance of supernatants was measured at 540 nm. Hemolysis (H) was calculated according to the formula:

H (%) = (A_sample_ − A_negative control_)/(A_positive control_ − A_negative control_) × 100 (1)

where A_sample_, A_negative control_, and A_positive control_ represent the absorbances of the tested sample, the negative control (erythrocytes in PBS buffer) and the positive control (erythrocytes in distilled water), respectively.

## 3. Results and Discussion

### 3.1. The Development of the Synthesis Methodology of Albumin Particles

In order to verify the most favorable synthesis parameters of albumin nanoparticles, a number of syntheses under various conditions were performed. The variable parameters with the observations accompanying the conditions applied are presented in Table 1. 

In the initial syntheses, a constant protein concentration was used. The albumin solvent as well as the type and the concentration of the salting-out agent were changed. First salting-out processes were conducted using the Tris-HCl buffer with pH = 4.8. In the case of the use of calcium chloride as a salting-out agent no effect was demonstrated. The protein solubility depends on many factors including the ionic strength of the solvent—as the ionic strength increases, the protein solubility decreases. It may be concluded that the use of the solution with higher ionic strength promotes the protein fractionation [37]. The calcium chloride solution used during the synthesis showed two times lower ionic strength than the potassium phosphate solution of the same concentration. As a result, an insufficient value of the ionic strength of the CaCl_2_ solution may result in the fact that the protein salting-out process did not take place (samples: 1, 2).

Next, despite the relatively high ionic strength of the potassium dihydrogen phosphate solution, the positive effect was also not observed. The reason is probably the fact that during the dissociation of the hydrogen salts hydrogen ions were formed which, in turn, interact less intensively with water than the metal ions (K^+^ cations) [38]. As a result, the potassium dihydrogen phosphate does not cause the protein salting-out process, which is probably due to the insufficient amount of potassium ions interacting strongly with water (samples: 3, 4).

Considering the process performed using K_3_PO_4_, the precipitation of proteins in a form of large agglomerates was observed. It is assumed that K_3_PO_4_ showed effective salting-out properties due to the high ionic strength of its solution and the potassium ions showing an ability to produce strong interactions with water dipoles (samples: 5, 6).

In the next syntheses, the albumin solution in Tris-HCl buffer with pH = 7 was used. The same salting-out agents, i.e., potassium phosphate, potassium dihydrogen phosphate and calcium chloride, were applied. The use of both calcium chloride and potassium dihydrogen phosphate still did not provide any effect in the next performed experiments (samples: 7, 8, 9, 10). In the case of the use of potassium phosphate and the Tris-HCl buffer with higher pH value a formation of noticeable cloudy mixture was observed (samples: 11, 12).

**Figure 2:**
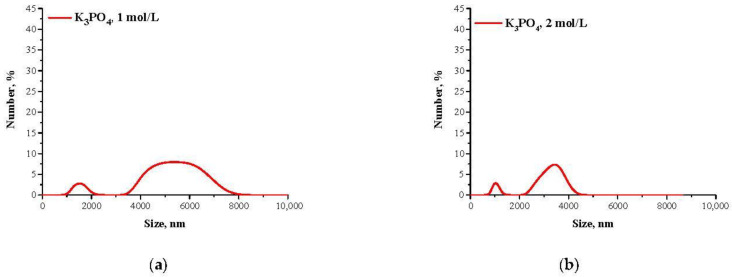
DLS analysis of particles obtained using Tris-HCl buffer with pH = 4.8 and 1 mol/L K_3_PO_4_ (**a**) or 2 mol/L K_3_PO_4_ (**b**) as a salting-out agent.

**Figure 3:**
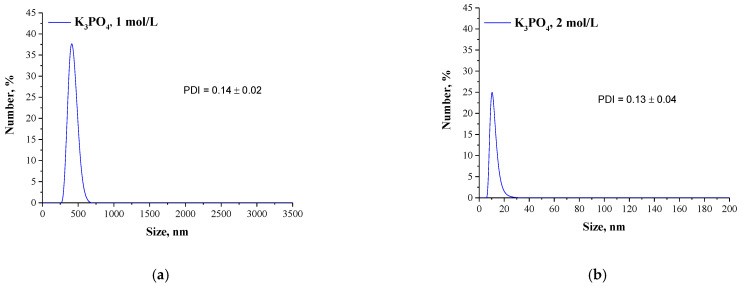
DLS analysis of particles obtained using Tris-HCl buffer with pH = 7 and 1 mol/L K_3_PO_4_ (**a**) or 2 mol/L K_3_PO_4_ (**b**) as a salting-out agent.

Mixtures, in which the protein precipitation was noticed, were subjected to the DLS analysis aimed at determining the size and the dispersity of obtained particles. Results of the measurements are presented in Figure 2 and Figure 3.

Due to the presence of ionizable functional groups in proteins, a formation of hydrogen bonds between the protein and the water molecules having a nature of dipoles occur. This, in turn, results in the formation of a characteristic water shell on the protein surface, defined as a hydration shell. The presence of such a shell prevents the protein attraction followed by their aggregation so the hydration shell is responsible for increasing the protein solubility. The hydration degree depends on the protein charge which is closely related to the pH of the solution in which such a protein occurs. The smallest hydration shell occurs at the pH value corresponding to the pI value because in such a situation the protein molecule has a net charge of 0 so it shows the lowest ability to interact with water. As a result, the proteins at pI have the highest tendency to attract and form agglomerates which, in turn, may easily precipitate out from the solution [39,40,41]. 

The described effect corresponds to the synthesis conditions when a buffer with pH value close to the pI of the albumin was applied. As a result, the particles obtained are characterized by large sizes and polydispersity, which is presented in Figure 2. The application of Tris-HCl buffer with pH different than the pI caused the protein molecules having a charge to interact more intensely with molecules of water and it was more difficult to separate them. Finally, homogeneous protein particles with small sizes were obtained, which is presented in Figure 3.

**Figure 4:**
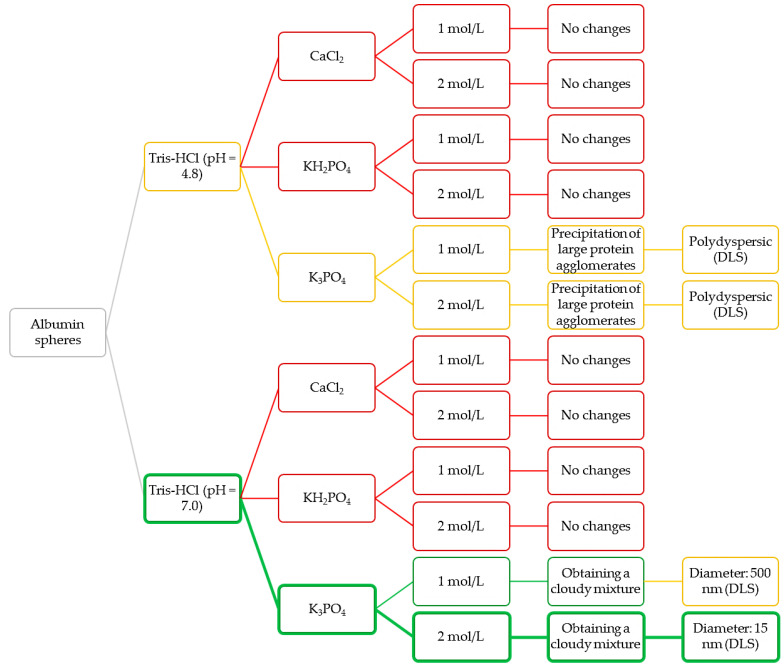
Design of the performed experiments aimed at selecting the most appropriate conditions for preparation of albumin nanoparticles.

Nanometric albumin particles show a great application potential. The synthesis of such nanomaterials is particularly interesting when they are considered as potential drug carriers. Carriers in a form of nanoparticles allow to improve the effectiveness of treatment and, importantly, reduce possible side effects [42]. They also show a very high ability to penetrate the cell membranes which, in turn, allows them to transport various types of active substances into the cells [43]. In the above-described connection, the purpose of the next step of the research was to verify the possibility of the synthesis of albumin nanoparticles using appropriately selected conditions. Below in Figure 4 a scheme of the Experimental Design of the performed studies aimed at selecting the pH of Tris-HCl buffer as well as the type and the concentration of the salting-out agent used for the synthesis of albumin particles are presented.

Based on the observations as well as on the results of DLS analyses, the Tris-HCl buffer with pH = 7 and the potassium phosphate as a salting-out agent (with concentration 2 mol/L) were selected for further experiments (green route of the Experimental Design). Next, syntheses aimed at determining the dependance between the concentration of albumin and the salting-out agent and the sizes of the albumin particles was obtained.

The conditions applied are presented in Table 2.

**Figure 5:**
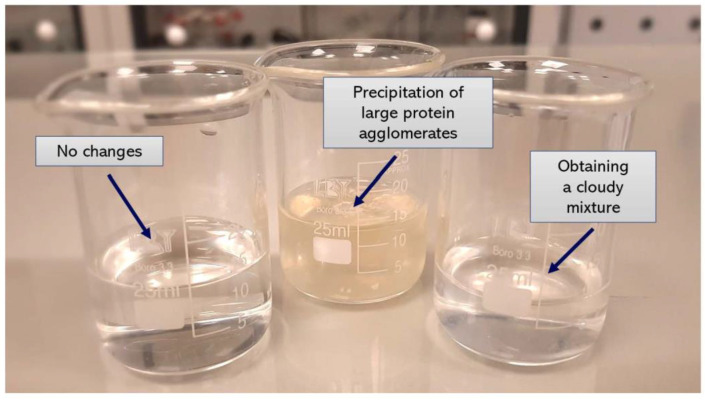
Examples of the compositions prepared.

Example suspensions obtained as a result of the performed syntheses are presented below in Figure 5.

Firstly, a constant concentration of potassium phosphate (i.e., 2 mol/L) was applied wherein the increasing protein concentration was used and finally the samples with the following ratios of potassium phosphate: albumin were prepared: 1:0.25; 1:0.5; 1:1; 1:2; 1:3 and 1:4. In the next syntheses, a constant albumin concentration and increasing concentrations of the salting-out agent were used. The albumin particles syntheses were performed via two methodologies, i.e., using a burette and by means of a syringe system. Detailed discussion of the results of DLS and FT-IR analyses is presented in the following sections of the paper. 

### 3.2. Investigations of the Albumin-Based Particles

#### 3.2.1. Measurements of Particle Sizes via DLS Method

**Figure 6:**
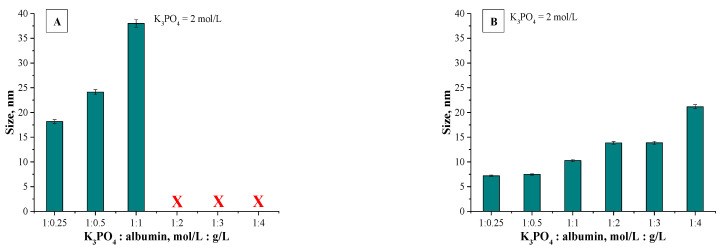
Results of DLS analysis of albumin particles obtained using various albumin concentrations via a syringe system (**A**) and using a burette (**B**) (*n* = 3, *n*—Number of repetitions).

**Figure 7:**
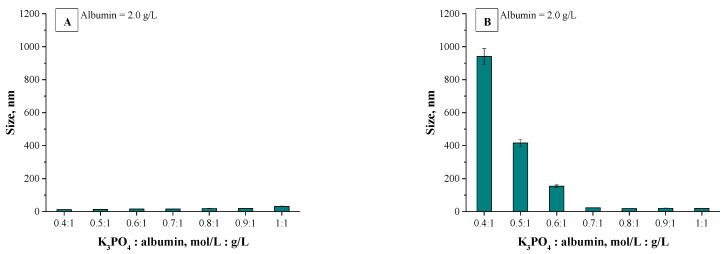
Results of DLS analysis of albumin particles obtained using various K_3_PO_4_ concentrations via a syringe system (**A**) and using a burette (**B**) (*n* = 3, *n*—Number of repetitions).

The application of the dynamic light scattering (DLS) technique allowed to precisely determine the sizes of the albumin-based particles. The average values from the performed measurements are presented below in Figure 6 (results for particles obtained using various albumin concentrations) and in Figure 7 (results for particles synthesized using various concentrations of the salting-out agent (i.e., K_3_PO_4_)).

Below in Table 3. values of the polydispersity index (PDI) of all prepared samples are listed.

Analyzing the above-presented results may lead to the conclusion that both the concentration of albumin and the salting-out agent affected the sizes of prepared albumin-based particles. In the case of particles obtained using various protein concentration it may be observed in Figure 6A,B that this variable had a meaningful impact on the diameter of the particles. With the increasing albumin concentration, the particles with larger diameter are obtained. In the case of the syntheses performed by means of the methodology with a burette, all particles prepared using a constant salting-out agent concentration and various albumin concentrations showed a nanometric size, i.e., between 5–25 nm. Moreover, the results obtained indicated the low size distribution, which may be evidence of preparing a relatively homogeneous suspension of albumin-based particles. Such an effect may be a result of the use of potassium phosphate with a higher molar concentration which, in turn, caused a high ionic strength of such formed suspension, influencing the process of salt induced protein precipitation.

Other conclusions may be drawn analyzing the results for particles obtained via a syringe system. In Figure 6A polydisperse suspensions with a very high size distribution were marked using a red “X”; such cases significantly impede a conclusive determination of an average particle size. Here, the particles obtained exhibited significantly larger sizes which were not within the nano range. It was concluded that this synthesis methodology (via a syringe system) led to the preparation of particles with high polydispersity and their suspension was highly inhomogeneous. The highest protein concentration providing the suspension monodispersity was obtained when the synthesis was performed using reagents in ratio 1:1. The use of higher protein content compared to the K_3_PO_4_ content led to the synthesis of particles which, due to their inhomogeneity, were not suitable from the viewpoint of their potential applicability.

Different conclusions were drawn in the case of albumin-based particles obtained using a constant albumin concentration and various K_3_PO_4_ concentrations. Results for such materials are shown in Figure 7A. Here, a polydispersity of prepared suspensions was not observed. Furthermore, all particles show nanometric sizes—their size is approx. 20 nm. It can be concluded that by applying a synthesis methodology based on a syringe system, the changing potassium phosphate concentration did not significantly affect the size of the particles obtained. The interesting results were obtained in the case of samples prepared using various potassium phosphate concentrations and via a burette (Figure 7B). It was stated that with an increasing amount of the salting-out agent concentration, the size of the particles obtained was lower. The highest sizes were noticed for particles prepared using the potassium phosphate solution and the albumin solution in 0.4:1 ratio.

Next, applying the K_3_PO_4_ solution and the albumin solution in 0.7:1 ratio and higher content of potassium phosphate a significant reduction in the size of the particles was reported. The dependance between the size of the particles obtained and the K_3_PO_4_ concentration results probably from the ionic strength of the salting-out agent solution. As the concentration of potassium phosphate increases, the ionic strength of such a solution increases and thus the salt induced protein precipitation proceeds more effectively. After exceeding a certain concentration of a salting-out agent, the ionic strength of such a solution is sufficiently high and makes it possible to obtain albumin-based particles within the nanometric sizes. Furthermore, for all particles obtained using a burette, results of DLS analysis indicate the single band. This, in turn, confirms the preparation of albumin-based particles with homogeneous sizes and, importantly, indicates that such a method allows to obtain albumin particles with strictly defined sizes closely correlated with parameters such as the concentration of the salting-out agent and the protein concentration.

#### 3.2.2. Morphological Analysis Using Transmission Electron Microscopy (TEM)

**Figure 8:**
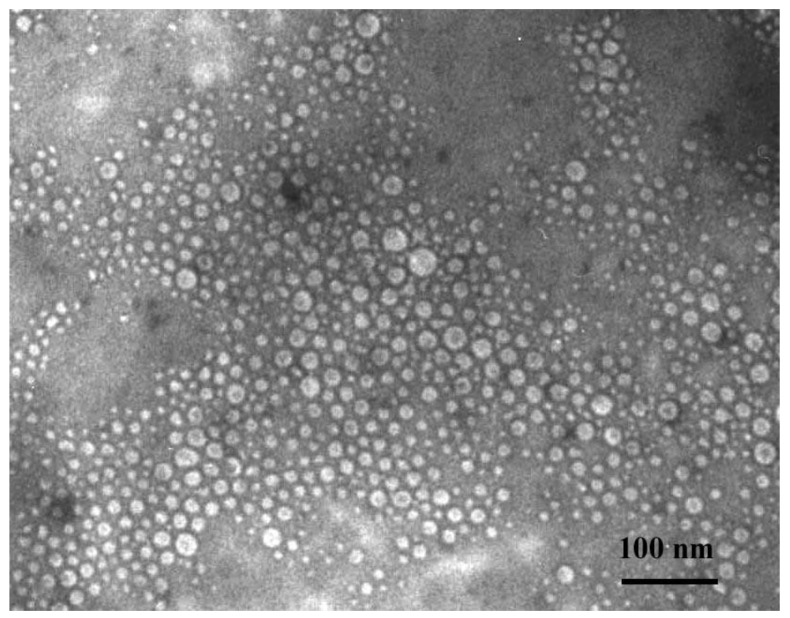
TEM Microphotograph of obtained albumin spheres (potassium phosphate concentration: Albumin concentration ratio—1:2 [mol/L: g/L]).

In Figure 8 results of the morphological analysis are presented.

Simultaneously the TEM analysis for model nanoparticles (sample 4) was performed to estimate the morphology of obtained materials. The prepared nanoparticles presented spherical shapes (Figure 8). Moreover, size of the tested albumin nanoparticles determined via the TEM analysis correlated well with the results obtained using the dynamic light scattering method.

#### 3.2.3. Analysis of Albumin Particles via FT-IR Spectroscopy

**Figure 9:**
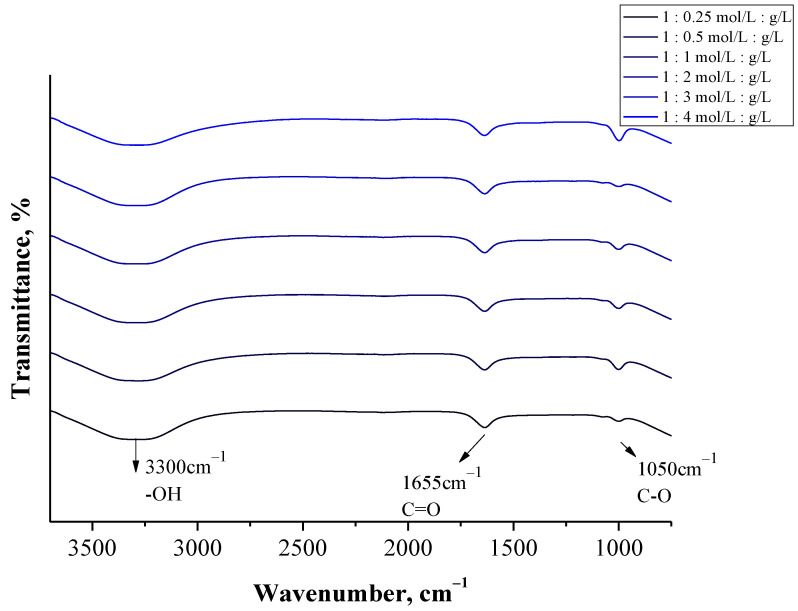
FT-IR spectra of particles obtained using a burette (at various albumin concentrations).

Figure 9 shows examples of the FT-IR spectra—spectra of albumin particles obtained via a burette (using a constant concentration of the salting-out agent and various albumin concentrations) are presented. (FT-IR spectra of the remaining particles are presented in Figures S1–S3 in the Supplementary File).

In the case of all tested samples, a wide band in the range of 3500–3000 cm^−1^ was observed, which may be assigned to the stretching vibrations of the OH groups [44]. Next, the absorption band at approx. 1050 cm^−1^ was identified on all FT-IR spectra. This band probably derives from the stretching vibrations of the C-O group [45]. Particular attention was paid to the bands within the range 1700–1500 cm^−1^. This is the region in which, in the case of proteins, absorption bands related to their secondary structure are expected [46]. On the FT-IR spectra of all analyzed samples an absorption band at approx. 1655 cm^−1^ was observed, which may be assigned to the C-O stretching mode from the amide I band. Moreover, in the case of some samples, the presence of an absorption band at 1543 cm^−1^ (Figure S3) corresponding to the C-*n* stretching vibrations coupled with *n*-H bending modes of amide II band may also be observed [47]. Both amide I and amide II bonds are characteristic for the functional groups forming the peptide bond between the amino acids included in the albumin.

The identified FT-IR bands indicated the presence of functional groups characteristic for proteins. Moreover, the bands identified are analogous to the results of similar studies presented in other publications where functional groups characteristic for albumin were also evidenced within the range of 1700–1500 cm^−1^ [48,49]. Thus, FT-IR spectroscopy allowed to confirm the preparation of relatively pure albumin particles.

#### 3.2.4. Results of the Investigation on the Cytotoxicity of Albumin Particles via MTT Reduction Assay

**Figure 10:**
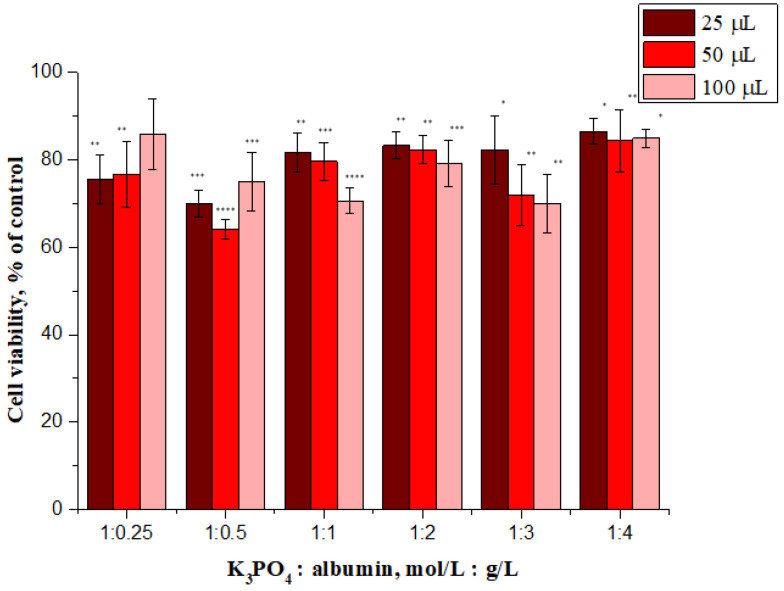
In vitro cytotoxicity of albumin nanoparticles (*samples 1–6*) to NHDF cells after incubation at 48 h. * show the statistical significance calculated with one-way analysis of variance (ANOVA) and the Dunnet post hoc test (* *p* ≤ 0.05, ** *p* ≤ 0.01, *** *p* ≤ 0.001 and **** *p* ≤ 0.0001), as compared to control (not treated cells).

Thus, in order to gain further overview of possible cytotoxic effects of synthesized nanoparticles, an activity screen in normal human dermal fibroblasts representing healthy cells was performed. Fibroblasts cells are often selected as exemplary control lines to demonstrate safety for various types of formulations [50,51,52]. The impact of albumin particles (concentrations of 25, 50 and 100 µL/mL) on the viability of NHDF cells was measured with the MTT assay after incubation for 48 h. Results of the performed investigations are shown in Figure 10.

Evaluation of cytotoxicity of tested materials is essential from the viewpoint of their potential application for biomedical purposes.

We also consider results presented in other investigations, for example, Salehiabar et al. described curcumin loaded bovine serum albumin nanoparticles synthesized by a simple coacervation procedure. Cellular toxicity of nanoparticles was investigated on normal human fibroblast cell lines (HFF-2). The MTT test showed that blank bovine serum albumin nanoparticles were highly biocompatible and do not possess a toxic effect (the determined cell viability >100%) [53]. Next, the same cell line was also used for cytotoxicity assessment for chrysin loaded bovine serum albumin nanoparticles, synthesized by a desolvation procedure, as reported by Nosrati et al. In addition, in this case, a very high level of viability of these cells (>100%) was noted for nanoparticles that were not loaded [54]. In another publication, colistin-loaded human albumin nanoparticles were prepared by a purposely tuned double-emulsion method and tested on a human foreskin fibroblasts cell line. The safety of the developed nanoformulation was demonstrated by a negligible cytotoxicity (cell viability was slightly less than 100%, and in some cases even greater) [55].

The treatment of the tested cell line with sample 1:1, sample 1:2 and sample 1:3 resulted in a dose-dependent decrease of cell viability whereas the highest viability (above 84%) was observed after treatment with sample 1:4 at all tested concentrations. In general, it is widely accepted that a cytotoxicity above 80% indicates non-toxicity, therefore this sample can be considered a non-toxic material. 

One possible explanation for this is that sample 1:4 has the largest size. As previously reported by Avalos et al., the toxicity of silver nanoparticles depends on the size; the smaller particles were significantly more cytotoxic than the larger particles to NHDF cells [56]. Therefore, it is possible that the trend we observed is a consequence of the above described effect. However, it should be emphasized that the changes in cytotoxicity are not large and further work is needed to explain the mechanism responsible for this phenomenon. It may be concluded that sample 1:4 meets the definition of a non-toxic material and is biocompatible when used at tested concentrations.

#### 3.2.5. Analysis of the Hemolytic Activity of Albumin Particles

In the next study, main attention was paid to the blood compatibility determination due to the fact that potential damage to erythrocyte membranes may constitute a serious obstacle, e.g., in the case of intravenous administration of developed formulations. Thus, to evaluate the safety of the newly fabricated nanoparticles, the hemolysis assay was performed. In this hemocompatibility test, albumin nanoformulations (at a concentration of 10 µL/mL) were incubated with isolated human red blood cells and hemolytic activity was assessed after measurement of hemoglobin released from erythrocytes at λ = 540 nm. For all tested samples (1–6), the calculated hemolysis did not exceed 2.5%, suggesting that developed albumin nanoparticles used at this dose could be considered as nonhemolytic material. The results obtained, indicating the lack of hemolytic properties of albumin-based nanoparticles, are consistent with other results obtained for human serum albumin [57,58] or bovine serum albumin [54] nanoparticles also confirming their non-toxicity to red blood cells. Thus, it was proved that albumin nanoparticles prepared using the potassium phosphate solution and the albumin solution in 1:4 ratio (sample 6) are the most promising formulation for biomedical applications due to low cytotoxicity and excellent blood biocompatibility.

## 4. Conclusions

The proposed salt-induced precipitation process is a method which allows to obtain polymer spheres. The selection of synthesis conditions such as the concentration of protein applied as well as the concentration and the type of the salting-out agent enables synthesis of albumin particles of various sizes. 

The use of salting-out agents such as CaCl_2_ or KH_2_PO_4_ did not allow to prepare albumin particles. This was probably caused by the relatively low ionic strength in the case of CaCl_2_ solution or by the presence of weakly interacting hydrogen ions in the case of KH_2_PO_4_ solution.

In the case of the salt-induced precipitation process performed using a burette and 2 M solution of K_3_PO_4_ as a salting-out agent, albumin spheres having a nanometric size (i.e., within the range 5–25 nm) were obtained, regardless of the protein concentration applied.

The size of the particles obtained depended both on the concentrations of the reagents used and the synthesis methodology applied. In the case of the syntheses carried out by means of a burette it was proved that as the concentration of the salting-out agent (K_3_PO_4_) increased, the diameter of the spheres obtained decreased. This was probably related to the increase in the ionic strength of the reaction mixture that favored the salt-induced protein precipitation process. In the case of the use of a syringe system, an increase in the K_3_PO_4_ concentration did not significantly affect the size of the particles obtained—in the case of all proposed conditions spheres having a size approx. 20 nm were obtained.

In the case of samples obtained using various protein concentrations it was proved that the synthesis methodology with a syringe system led to the preparation of particles of large polydispersity. The highest albumin concentration which allowed to prepare homogeneous particles was 2 g/L; above this concentration the particles obtained were heterogeneous and highly polydisperse.

The size of the nanospheres obtained as well as their spherical shape were confirmed via TEM analysis. Moreover, such results were consistent with results of DLS measurements.

The presence of the functional groups such as -OH and -NH_2_ characteristic for amino acids—main components of proteins—was verified via FT-IR analysis. In the case of highly polydisperse samples with large protein agglomerates an additional absorption band at 1543 cm^−1^, characteristic for amide II bonds, was reported.

Regarding the obtained UV-Vis spectra, a maximum absorbance at a wavelength of 288 nm was observed, which is characteristic for aromatic amino acids such as tryptophan included in the structure of albumin.

Performed cytotoxicity investigations on the normal human dermal fibroblasts showed that the highest cell viability—i.e., above 84%—was determined for samples obtained using potassium phosphate solution (2.0 mol/L) and albumin solution in 1:4 ratio. Moreover, all samples showed no hemolytic activity toward human erythrocytes. In the case of all samples, the hemolysis calculated did not exceed 2.5%. 

Due to the very promising results of biological investigations, the main focus during the next studies will be on the introduction of the active substance (i.e., cytostatic drug) into the developed albumin particles and the analyses using cancer cells.

## Figures and Tables

**Table 1:** The syntheses conditions of albumin-based carriers at a constant albumin concentration (2.0 g/L).

Sample	Albumin Solvent	Salting-Out Agent	Salting-Out Agent Concentration [mol/L]	Observations
1	Tris-HCl (pH = 4.8)	CaCl_2_	1	No changes
2			2	No changes
3		KH_2_PO_4_	1	No changes
4			2	No changes
5		K_3_PO_4_	1	Precipitation of large protein agglomerates
6			2	Precipitation of large protein agglomerates
7	Tris-HCl (pH = 7)	CaCl_2_	1	No changes
8			2	No changes
9		KH_2_PO_4_	1	No changes
10			2	No changes
11		K_3_PO_4_	1	Obtaining a cloudy mixture
12			2	Obtaining a cloudy mixture

**Table 2:** The synthesis conditions of albumin-based carriers with varying concentrations of albumin and salting-out agent solutions.

**Sample**	Potassium Phosphate Concentration *: Albumin Concentration RATIO **(mol/L: g/L)**
1	1:0.25
2	1:0.5
3	1:1
4	1:2
5	1:3
6	1:4
**Sample**	Potassium Phosphate Concentration: Albumin Concentration ** RATIO **(mol/L: g/L)**
7	0.4:1
8	0.5:1
9	0.6:1
10	0.7:1
11	0.8:1
12	0.9:1
13	1:1

* at a constant concentration of potassium phosphate = 2.0 mol/L; ** at a constant concentration of albumin = 2.0 g/L.

**Table 3:** PDI values of prepared samples.

**Sample**	Potassium Phosphate Concentration *: Albumin Concentration RATIO **(mol/L: g/L)**	**PDI** **(Syringe System)**	**PDI** **(Burette)**
1	1:0.25	0.13 ± 0.021	0.09 ± 0.024
2	1:0.5	0.15 ± 0.015	0.10 ± 0.016
3	1:1	0.12 ± 0.018	0.13 ± 0.011
4	1:2	0.85 ± 0.022	0.14 ± 0.012
5	1:3	0.79 ± 0.013	0.08 ± 0.022
6	1:4	0.98 ± 0.017	0.06 ± 0.014
**Sample**	Potassium phosphate concentration: Albumin concentration ** RATIO **(mol/L: g/L)**		
7	0.4:1	0.18 ± 0.018	0.16 ± 0.011
8	0.5:1	0.13 ± 0.022	0.13 ± 0.018
9	0.6:1	0.14 ± 0.011	0.09 ± 0.023
10	0.7:1	0.17 ± 0.017	0.11 ± 0.022
11	0.8:1	0.20 ± 0.023	0.12 ± 0.010
12	0.9:1	0.19 ± 0.014	0.13 ± 0.013
13	1:1	0.12 ± 0.019	0.13 ± 0.019

* at a constant concentration of potassium phosphate = 2.0 mol/L; ** at a constant concentration of albumin = 2.0 g/L.

## Data Availability

Data sharing is not applicable for this article.

## Supplementary Materials

The following are available online at https://www.mdpi.com/article/10.3390/ma14164386/s1. Figure S1: FT-IR spectra of particles obtained using a burette (at various salting-out agent concentration), Figure S2: FT-IR spectra of particles obtained using a syringe system (at various salting-out agent concentration), Figure S3: FT-IR spectra of particles obtained using a syringe system (at various albumin concentration), Figure S4: Results of UV-Vis analysis of albumin particles obtained using a burette (a) and via a syringe system (b).
