# Supplementary material for: The Development of the Innovative Synthesis Methodology of Albumin Nanoparticles Supported by Their Physicochemical, Cytotoxic and Hemolytic Evaluation
Source: Materials (Basel). 2021 Aug 5;14(16):4386. doi: 10.3390/ma14164386 (PMC8400698; doi:10.3390/ma14164386)
Supplement: Supplementary file 1 [file materials-14-04386-s001.zip › materials-1298111-supplementary.pdf]

materials

MDPI

Supplementary Material

The Development of the Innovative Synthesis Methodology of Albumin Nanoparticles Supported by their Physicochemical, Cytotoxic and Hemolytic Evaluation

Sonia Kudłacik-Kramarczyk [1,*], Anna Drabczyk [1,*], Magdalena Głąb [1,*], Paweł Gajda [2], Anna Czopek [3], Agnieszka Zagórska [3], Anna Jaromin [4], Jerzy Gubernator [4], Agnieszka Makara [5] and Bożena Tyliszczak [1,*]

[1] Department of Materials Science, Faculty of Materials Engineering and Physics, Cracow University of Technology, 37 Jana Pawła II Av., 31-864 Krakow, Poland
[2] Department of Sustainable Energy Development, Faculty of Energy and Fuels, AGH University of Science and Technology, 30 Mickiewicza Av., 30-059 Krakow, Poland; pgajda@agh.edu.pl
[3] Department of Medicinal Chemistry, Faculty of Pharmacy, Jagiellonian University Medical College, 9 Medyczna St., 30-688 Krakow, Poland; anna.czopek@uj.edu.pl (A.C.); agnieszka.zagorska@uj.edu.pl (A.Z.)
[4] Department of Lipids and Liposomes, Faculty of Biotechnology, University of Wroclaw, 14a Joliot-Curie St., 50-383 Wroclaw, Poland; anna.jaromin@uwr.edu.pl (A.J.), jerzy.gubernator@uwr.edu.pl (J.G.)
[5] Faculty of Chemical Engineering and Technology, Cracow University of Technology, 24 Warszawska St., 31-155 Krakow, Poland; agnieszka.makara@pk.edu.pl
* Correspondence: sonia.kudlacik-kramarczyk@pk.edu.pl (S.K.-K.); anna.drabczyk2@pk.edu.pl (A.D.); magdalena.glab@doktorant.pk.edu.pl (M.G.); bozena.tyliszczak@pk.edu.pl (B.T.)

Results of FT-IR spectroscopy of prepared albumin particles

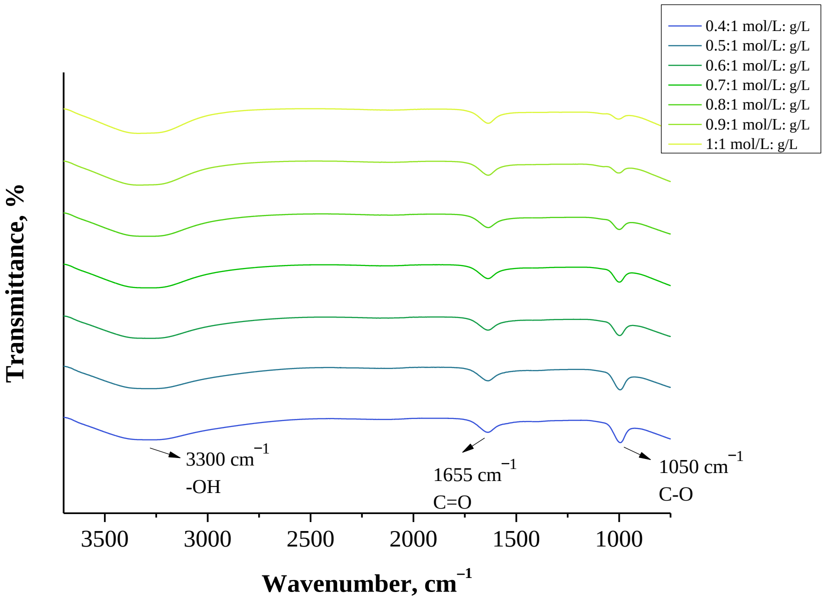

Figure S1. FT-IR spectra of particles obtained using a burette (at various salting-out agent concentration).

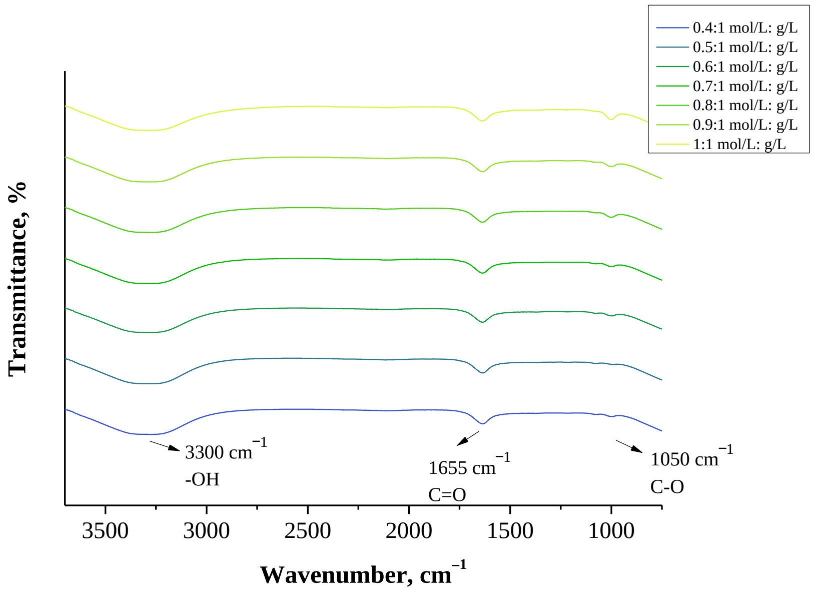

Figure S2. FT-IR spectra of particles obtained using a syringe system (at various salting-out agent concentration).

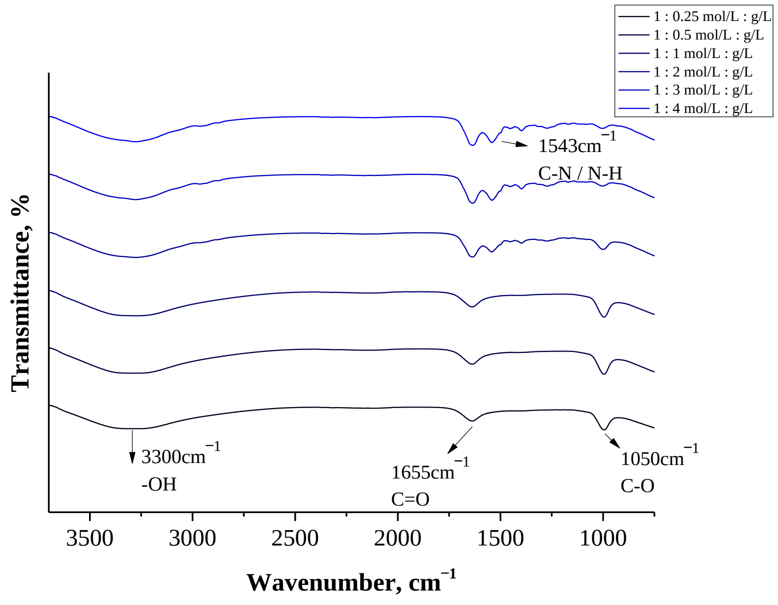

Figure S3. FT-IR spectra of particles obtained using a syringe system (at various albumin concentration).

Results of the UV-Vis spectrometry

Obtained UV-Vis spectra are presented below in Figure S4. Results are presented separately for each synthesis route.

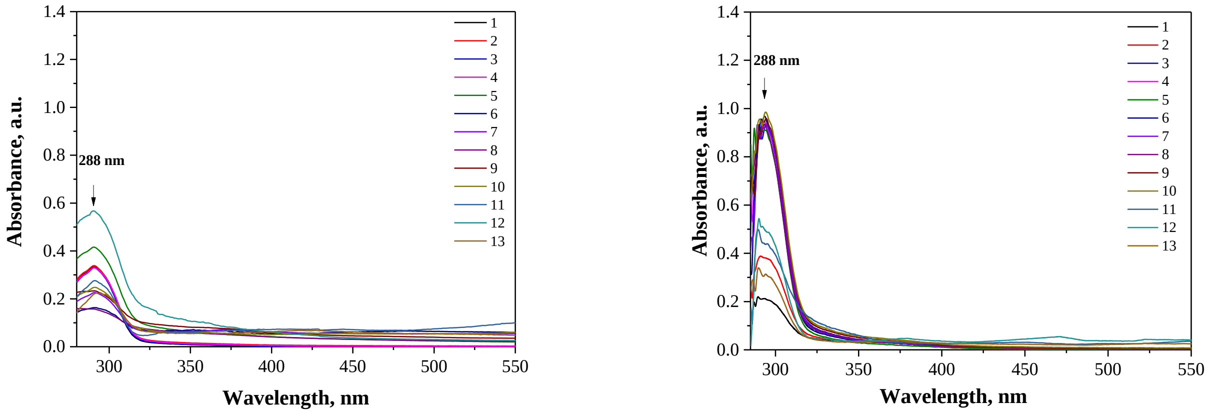

(**a**) (**b**)

Figure S4. Results of UV-Vis analysis of albumin particles obtained using a burette (**a**) and via a syringe system (**b**).

The fluorescent and absorptive properties of proteins result from the amino acid residues present in their structures. In the case of albumin, such residues are formed by tryptophan (Trp), phenylalanine (Phe) and tyrosine (Tyr). A common feature of these structures are aromatic rings which are able to absorb UV light. All amino acids exhibit maximum absorption at a specific wavelength range. These compounds show also different values of the molar absorption coefficient.

As it may be seen in the above figures, the maximum absorbance at 288 nm for all tested albumin particles was observed. Such a band indicated an overlapping maximum absorbance of tyrosine and tryptophan. Moreover, this absorption resulted probably from the $\pi \rightarrow \pi^*$ transitions of phenyl rings present in the structures of aromatic amino acids. The slight shifts of the absorption maxima depending on the type and the pH of the solvent applied as well as on the conformation of the tested protein may be observed in the absorption spectra of the aromatic amino acids.
